# Effects of dietary black soldier fly larvae meal inclusion on the serum metabolome of Silkie crossbreed chickens

**DOI:** 10.3389/fvets.2026.1803699

**Published:** 2026-04-01

**Authors:** Cheuk Ming Li, Ákos Kenéz

**Affiliations:** Department of Infectious Diseases and Public Health, City University of Hong Kong, Kowloon, Hong Kong SAR, China

**Keywords:** black soldier fly larvae, chickens, growth performance, gut health, lipid metabolism, serum metabolomics

## Abstract

**Introduction:**

Black soldier fly larvae (BSFL; *Hermetia illucens*) meal, an alternative to conventional dietary protein sources, such as soybean meal, is rich in medium-chain fatty acids, antimicrobial peptides and other bioactive components. These components may modulate the composition and function of the gut microbiota or, after absorption, affect host metabolic pathways. We hypothesised that the functional effects of BSFL meal would be reflected in alterations in the serum metabolite profile of Silkie crossbreed chickens.

**Methods:**

Serum samples from chickens fed a control soybean-based diet or a diet containing 150 g/kg partially defatted BSFL meal were analysed using untargeted liquid chromatography–mass spectrometry.

**Results and Discussion:**

A total of 3304 metabolite features were detected, of which 1341 were annotated, with 777 retained for statistical analysis. Principal component analysis and PERMANOVA revealed a clear separation between dietary groups (*p* = 0.001). Forty metabolites were significantly altered (FDR ≤ 0.05), with 31 more abundant and nine less abundant in the BSFL group. These shifts were characterised mainly by increased lipid-related metabolites, including medium-chain fatty acids (MCFAs) and phospholipids, indicating alterations in lipid metabolism. In addition, changes in amino acid derivatives and energy-related metabolites suggested the modulation of amino acid and energy metabolic pathways. Several metabolites with putative microbial origin were elevated, consistent with indirect host–microbiome metabolic interactions. These metabolic changes showed correlations with significantly higher average daily gain (*p* = 0.02), numerically greater final live weight, and a reduced feed conversion ratio in BSFL-fed chickens (*p* = 0.07 and *p* = 0.08, respectively). Overall, dietary inclusion of 150 g/kg BSFL meal modified systemic metabolism without detectable negative effects on performance or serum biomarkers, suggesting that this level of BSFL inclusion can be beneficial for slow-growing Silkie chickens.

## 1 Introduction

In regions such as Southeast Asia, where slow-growing poultry breeds are predominant, integrating insect-based feeds, such as black soldier fly larvae (BSFL; *Hermetia illucens*), into livestock production can offer substantial environmental and economic benefits. BSFL consume organic waste and turn it into high-quality protein, thereby reducing the environmental footprint of poultry production (1, 2). Beyond their role in waste upcycling, BSFL have emerged as a promising alternative in poultry nutrition, valued for their high-quality protein and diverse bioactive compounds (3). Notably, BSFL-derived antimicrobial peptides disrupt pathogen membranes and enhance immune responses, whilest medium-chain fatty acids (MCFAs) selectively suppress harmful gut bacteria without disturbing beneficial commensals (4, 5). These functional components help stabilise the gut microbiota, improve nutrient absorption and fortify host defences (6). BSFL meal as a functional feed ingredient is valuable considering rising antimicrobial resistance, which is driven by prolonged antibiotic use and poses escalating threats to animal and public health (7).

Lauric acid (C12:0), a predominant MCFA in BSFL, plays a vital role in shaping the intestinal microbiota. Its targeted antimicrobial activity suppresses pathogens such as *Clostridium perfringens* and *Bacteroides* spp. whilest exerting minimal effects on beneficial microbes (8, 9). This microbiota modulation enhances gut barrier integrity and promotes efficient nutrient uptake, ultimately supporting systemic health and growth performance. These effects are especially advantageous for slow-growing poultry breeds, which often require more stable intestinal environments (10, 11).

Silkie chickens, an indigenous slow-growing breed widely valued in Asia for their distinctive black skin, unique plumage, and cultural significance, were selected as the experimental model in this study (12, 13). Compared to the predominant commercial broiler breeds such as Ross 308, Silkie chickens exhibit slower growth rates and smaller body size, and are prized for their meat quality in specialty markets. Their significant presence in the Southeast-Asian poultry sector makes them particularly relevant for evaluating sustainable feed ingredients, such as BSFL.

Despite these observations, the metabolic features underlying BSFL-induced improvements in poultry remain poorly understood. Bioactive components of BSFL meal, including MCFAs and antimicrobial peptides, may influence host metabolism either indirectly, by modulating the composition and functional activity of the gut microbiome, or directly, through absorption and interaction with host metabolic pathways. Therefore, we hypothesise that dietary inclusion of BSFL meal induces distinct shifts in serum metabolite profiles and alters metabolic pathways, which can help explain the previously documented dietary effects. Advancements in metabolomics have enabled the assessment of a broad range of metabolites, facilitating a deeper understanding of interactions between dietary treatments and metabolic features. Untargeted metabolomics–the comprehensive analysis of metabolites within a biological system–provides detailed snapshots of metabolic states and serves as a powerful tool to uncover biochemical responses to dietary interventions (14). Metabolomics has been successfully used to reveal diet-induced metabolic shifts in poultry (15, 16); however, the metabolic effects of BSFL-based diets have not yet been characterised, underscoring the value of applying metabolomics in this context.

In our previous study investigating the same cohort of Silkie chickens as the present research, the dietary inclusion of 150 g/kg BSFL meal increased average daily gain, reduced the feed conversion ratio and significantly reduced serum D-lactate, suggesting better gut barrier function (3). Building on these findings, the present study applies untargeted liquid chromatography–mass spectrometry (LC–MS)-based metabolomics to serum samples obtained from these chickens to identify metabolic shifts and pathway adaptations that may help explain our previously observed performance benefits. By characterising these metabolite shifts, this study seeks to reveal the biochemical mechanisms underlying the dietary effects and provide insights that may inform future feeding strategies for sustainable poultry production.

## 2 Materials and methods

### 2.1 Birds and sample collection

A total of 72 female Silkie crossbreed chickens (39 days of age) were randomly assigned to three dietary treatments in the original trial: a control group (CON) and two groups receiving BSFL meal at different inclusion levels. Birds were selected at 39 days to match the late grower phase, when dietary adjustments are typically implemented in slow-growing breeds, ensuring uniform pre-trial management and industry relevance (17). The BSFL meal was supplied by E Farm Biotech Ltd. (Hong Kong, China), a commercial producer, and was derived from larvae reared on a substrate consisting of bakery waste and soy pulp. After partial defatting, the meal contained 53% crude protein and 33% crude fat (DM basis), as determined by proximate analysis. Only the higher inclusion group was included in the present metabolomics study. Each group consisted of eight replicates, with three chickens per cage.

The CON diet consisted of 635 g/kg corn, 260 g/kg soybean meal, 25 g/kg soybean oil, 10 g/kg fish meal, 30 g/kg wheat bran and 40 g/kg vitamin–mineral premix. The BSFL diet replaced fish meal and soybean oil with 150 g/kg partially defatted black soldier fly larvae meal, and soybean meal was reduced to 145 g/kg, whilest the proportions of corn, wheat bran and premix remained unchanged. The diets were mixed in the farm’s in-house feed mill and formulated to provide similar energy levels. Metabolisable energy and proximate composition were calculated and analysed in the original parent study (3). The CON diet contained 890 g/kg dry matter, 206 g/kg crude protein and 16.8 MJ/kg metabolisable energy, whilest the BSFL diet contained 890 g/kg dry matter, 235 g/kg crude protein and 16.9 MJ/kg metabolisable energy (on a DM basis).

Clinical health and mortality were monitored daily, and body weight and feed consumption were recorded weekly throughout the experimental period (39–60 days of age). Average daily weight gain, and feed conversion ratio were calculated collectively for the study period. In addition to the summary herein, further details on the methodology and growth performance results are available in the original parent study (3). At the end of the experiment, blood samples were collected from two randomly selected birds (60 days of age) per cage. Blood was drawn from the brachial vein into 1.3 mL serum tubes (Sarstedt, Nümbrecht, Germany), and serum was separated by centrifugation at 10,000 × g for 5 min at room temperature. The resulting serum samples were stored at −80 °C until further analysis.

### 2.2 Untargeted metabolomics analysis of serum

Serum samples from the CON and BSFL groups, which previously showed significant differences in growth performance and serum D-lactate levels [3], were submitted to BGI Bio-solutions Hong Kong Co. Limited (Hong Kong) for untargeted metabolomics analysis to investigate differences in serum metabolite profiles. The analysis followed the BGI standard workflow for LC–MS. Metabolites were extracted using a mixture of methanol, acetonitrile and water (2,2,1, v/v/v) (18).

Chromatographic separation was performed on an ACQUITY UPLC BEH C18 column (1.7 μm, 2.1 mm × 100 mm; Waters Corporation, Milford, USA) using a Waters UPLC I-Class Plus system coupled to a tandem Q Exactive high-resolution mass spectrometer (Thermo Fisher Scientific, Waltham, USA). The column temperature was maintained at 45 °C. For positive ion mode, the mobile phase consisted of 0.1% formic acid (A) and acetonitrile (B). For negative ion mode, the mobile phase consisted of 10 mM ammonium formate (A) and acetonitrile (B). The gradient conditions were as follows: 0–1 min, 2% B; 1–9 min, 2–98% B; 9–12 min, 98% B; 12–12.1 min, 98% B to 2% B; and 12.1–15 min, 2% B. The final 2.9 min served as the column re-equilibration step between runs. The flow rate was set to 0.35 mL/min, with an injection volume of 5 μL.

Mass spectrometry was performed in both positive and negative ionisation modes using an electrospray isolation source. Spray voltages were 3.8 kV (positive-ion mode) and −3.2 kV (negative-ion mode), respectively. The capillary temperature was 320 °C, auxiliary gas heater temperature was 350 °C, sheath gas flow rate was 40, and auxiliary gas flow rate was 10. The full scan range was 70–1,050 m/z, with a resolution of 70,000, an AGC target of 3e6, and a maximum injection time of 100 ms. MS/MS spectra were acquired in data-dependent acquisition mode, selecting the top 3 precursors per cycle, with a resolution of 17,500, an AGC target of 1e5, a maximum injection time of 50 ms, and stepped normalised collision energies of 20, 40 and 60 eV. Quality control samples were prepared by pooling equal volumes from each serum sample and were interspersed every 10 samples.

Data preprocessing was carried out using Compound Discoverer v3.3 (Thermo Fisher Scientific) and metaX v2.0.0. to extract peaks and identify metabolites, obtaining peak areas and identification results. Parent ion mass tolerance was <5 ppm, fragment ion mass tolerance <10 ppm, and retention time alignment window <0.2 min. Metabolites with CV > 30% in QC samples were removed. Normalisation was performed using probabilistic quotient normalisation combined with QC-based robust LOESS signal correction. Further classification and functional annotation analysis of identified metabolites was conducted to obtain KEGG IDs, HMDB IDs, categories and KEGG pathways by comparison against public databases, including BMDB (BGI Metabolome Database), mzCloud, ChemSpider, HMDB, KEGG and LipidMaps (versions current at the time of analysis in 2024). Statistical analyses were performed in R v4.0.4.

### 2.3 Data analysis and visualisation

Metabolome data were analysed using MetaboAnalyst 6.0, following normalisation by sum, log transformation (base 10) and Pareto scaling (19). Prior to statistical analysis, metabolite features classified as Annotation Level 4 or Level 5 – namely, chemical signals without reliable structural annotations or only tentative matches to compound libraries – were excluded, and only those classified as Annotation Level 3 or above were retained, in alignment with the Metabolomics Standards Initiative reporting guidelines (20, 21). Principal component analysis (PCA) was employed to visualise the overall variation in the metabolome data, identifying patterns or outliers amongst the samples. The *t*-test was used to identify metabolite changes, and the resulting *p*-values were adjusted for multiple comparisons using false discovery rate (FDR) correction. Given the semiquantitative nature of untargeted metabolomics in the absence of authentic standards, metabolite changes were reported as log_2_ fold change (BSFL/CON); metabolites with FDR-adjusted *p*-values ≤0.05 and log_2_ fold change ≥1 or ≤−1 were considered statistically significant. The list of significant metabolites was subsequently analysed for pathway enrichment. Quantitative enrichment analysis was performed with sum normalisation, Pareto scaling and log transformation (base 10) to account for differences in overall signal intensity and variance structure. Pathway mapping was conducted against the Small Molecule Pathway Database. A heatmap illustrating correlations between the previously published serum metabolites and growth performance and gut health biomarkers (3) was generated in R using the *ggplot2* package, with significance levels based on adjusted *p*-values.

## 3 Results

### 3.1 Growth performance and serum indicators of gut health

Growth performance and serum biomarker data from the larger parent study were published previously (3), and the relevant data of the treatment groups included in the current metabolomics study are shown in Table 1. In summary, average body weight increased throughout the trial in both the CON and BSFL groups, with no statistically significant differences between these groups at any sampling point (*p* > 0.05). Both average daily weight gain (ADG) and feed conversion ratio (FCR) were significantly improved in the BSFL group during week 2 compared with the CON group (*p* = 0.01). ADG over the entire trial period was significantly higher in the BSFL group compared with the CON group (*p* = 0.02). Daily feed consumption did not differ significantly between the two groups (*p* = 0.77). FCR was numerically lower in the BSFL group compared with the CON group (*p* = 0.08). No clinically apparent diseases or mortality were observed throughout the experiment. The BSFL group tended to have lower serum LPS concentrations than the CON group (*p* = 0.09). Serum D-lactate concentrations were significantly reduced in the BSFL group compared with the CON group (*p* < 0.01). There was no difference in serum ALP concentrations between the CON and BSFL groups (*p* = 0.64).

**Table 1:** Effects of black soldier fly larvae meal inclusion on growth performance (body weight, average daily gain, daily feed consumption, and feed conversion ratio) and serum biomarkers (lipopolysaccharides, D-lactate, and alkaline phosphatase) in Silkie crossbreed chickens from 39 to 60 days of age.

Parameters	Day of age	CON	BSFL	SEM	*p*-value
Average body weight	39 d	607.5	619.38	7.37	0.44
	46 d	825.83	844.17	9.45	0.35
	53 d	1068.75	1118.33	14.60	0.09
	60 d	1,265	1342.5	21.75	0.07
Average daily weight gain	39–60 d	31.31^a^	34.43^b^	0.70	0.02
Daily feed consumption	39–60 d	240.98	242.99	3.41	0.77
Feed conversion ratio	39–60 d	2.61	2.39	0.06	0.08
Serum concentration (μg/mL)					
LPS		14.18	5.64	2.52	0.09
D-Lactate		41.01^a^	32.73^b^	1.46	<0.01
ALP		407.92	378.35	31.33	0.64

CON, control diet; BSFL, partial replacement of soybean meal with 150 g/kg partially defatted BSFL meal; SEM, standard error of the mean; LPS, lipopolysaccharides; ALP, alkaline phosphatase. Different superscripts indicate *p* < 0.05 based on *t*-test comparisons.

### 3.2 Serum metabolomic shifts induced by BSFL inclusion

**Figure 1:**
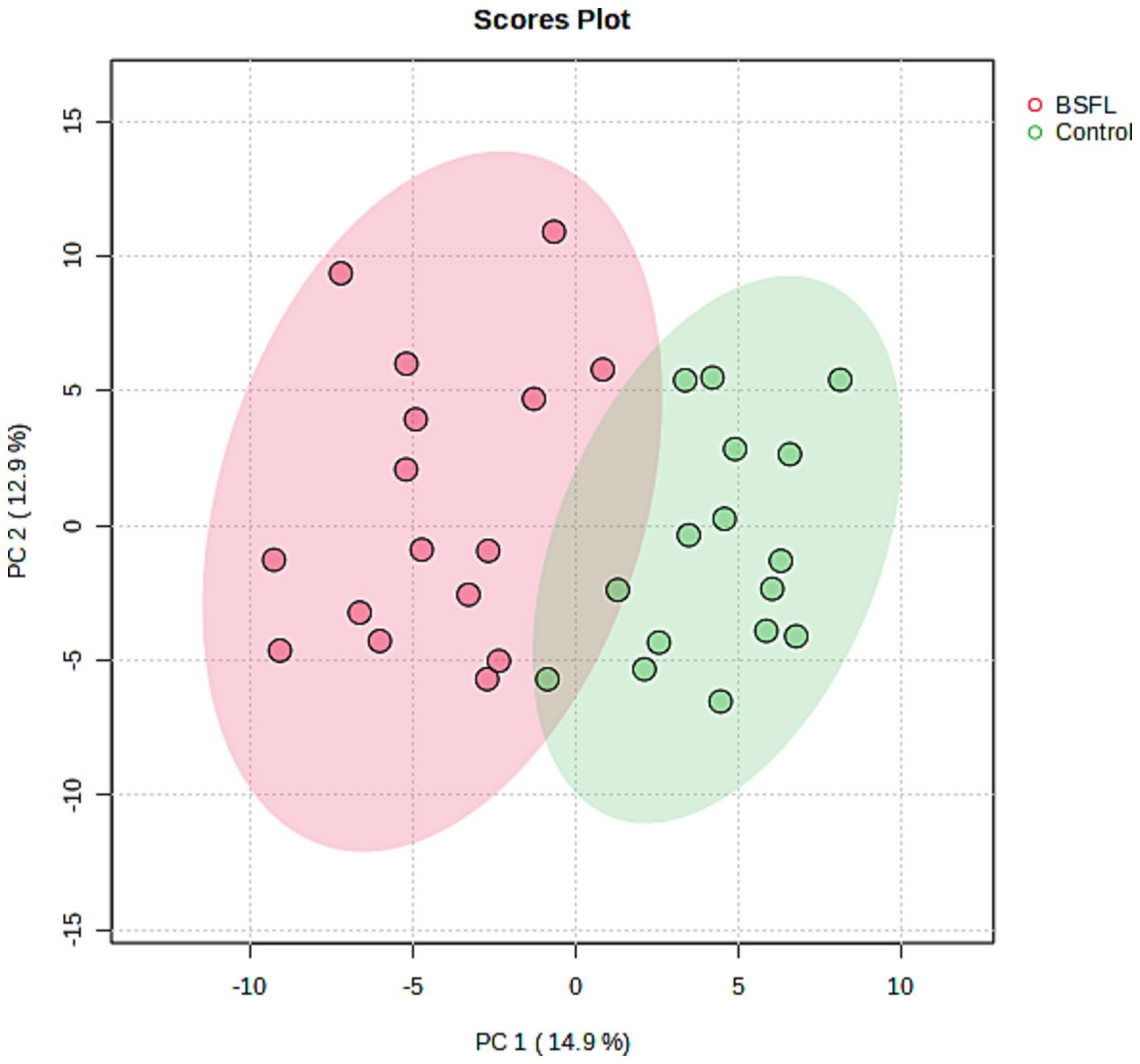
Scores plot of principal component analysis (PCA) between principal components 1 and 2 applied to the serum metabolome of chickens at the end of the trial. Each symbol represents the principal components of one chicken. Symbols with different colors indicate different dietary groups (CON vs. BSFL).

To address the impact of the BSFL inclusion diet compared to the control diet, the serum metabolome was analysed at the end of the trial. A total of 3,304 metabolite features were detected in the serum samples following data preprocessing. Of these, 1,341 metabolites were successfully annotated with identification information. For downstream statistical analysis, 777 metabolite features were retained. The PCA scores plot from the serum metabolome data demonstrated a significant separation between the BSFL diet group and the control group, as shown in Figure 1. The two principal components together accounted for 27.8% of the total variation, with PC1 contributing 14.9% and PC2 contributing 12.9%. This separation was further supported by PERMANOVA results, which yielded a statistically significant *p*-value of 0.001 based on 999 permutations. The *t*-test analysis identified 40 significantly different features out of 777 (FDR-adjusted *p* < 0.05, fold change ≥2) when comparing the BSFL diet group to the control group.

Untargeted metabolomic analysis identified 40 serum metabolites that were significantly different between the BSFL and CON groups (FDR-adjusted *p* ≤ 0.05). Of these, 31 were more abundant and 9 less abundant in the BSFL group. Log_2_ fold changes ranged from −2.84 to 7.71, indicating substantial shifts in circulating metabolite levels associated with dietary treatment (Table 2).

**Table 2:** Differentially abundant serum metabolites between BSFL and control diet groups identified by *t*-test with FDR-adjusted *p*-values ≤0.05 and log_2_ fold change.

Metabolites	Level of identification^#^	Log_2_ fold change^*^	FDR-adjusted *p*-value
Dimethyl sulfone	1	−2.84	<0.01
NP-000124 (C12H20O4)	3	−2.56	<0.01
4-Methylcatechol 2-sulphate	1	−1.79	<0.05
NP-018817 (C15H24O4)	3	−1.51	<0.01
NP-022010 (C14H23NO5)	3	−1.40	<0.05
14(S)-HDHA	3	−1.29	<0.01
Cyclohexylamine	2	−1.29	<0.01
2-Aminoheptanoate	3	−1.22	<0.01
Desthiobiotin	3	−1.22	<0.01
PE(16:0/18:1(9Z))	3	1.04	<0.01
Pyridoxamine	1	1.11	<0.01
Paracetamol sulphate	2	1.12	<0.05
4-Methylcatechol 1-sulphate	1	1.20	<0.05
Indole-3-carboxylic acid	2	1.29	<0.01
PC(14:0/20:4(5Z,8Z,11Z,14Z))	1	1.33	<0.01
Methyl N-[1-(4-fluorophenyl)ethylidene]-{[(dimethylamino)methylidene]amino}methanehydrazonothioate	3	1.75	<0.01
Myristic acid	1	1.78	<0.01
N-[3-(3-chloro-10,11-dihydro-5H-dibenzo[b,f]azepin-5-yl)propyl]-N, N-dimethylamine hydrochloride	3	1.82	<0.01
N-Acetylanthranilic acid	2	1.84	<0.01
1-(1-{[(3R,4S)-3-{[5-(Phenoxymethyl)-1,2-oxazol-3-yl]methyl}-4-piperidinyl]acetyl}-4-piperidinyl)-1,3-dihydro-2H-benzimidazol-2-one	3	1.89	<0.01
SM(d18:1/14:0)	1	1.92	<0.01
N-butyl-N′-[5-(tert-butyl)-1,3,4-thiadiazol-2-yl]urea	3	1.98	<0.01
LysoPC(14:0/0:0)	1	1.98	<0.01
p-Aminobenzoic acid	3	1.99	<0.01
PC(14:0/18:2(9Z,12Z))	1	2.02	<0.01
Zolazepam	3	2.05	<0.01
N-Methyl-N-{4-[2-(1-methylquinolinium-2-yl)vinyl]phenyl}methanamine	3	2.05	<0.01
(3S)-3-(5-Methoxy-1H-benzimidazol-2-yl)-N-phenyl-1-pyrrolidinecarboxamide	3	2.11	<0.01
methyl 2-cyano-3-{1-[3-(1H-imidazol-1-yl)propyl]-1H-pyrrol-2-yl}acrylate	3	2.15	<0.01
2-Amino-N-(1-methylethyl)benzamide	3	2.18	<0.01
PC(14:0/16:0)	3	2.49	<0.01
Lauric acid	1	2.70	<0.01
Trimethyl-5-aminovaleric acid	1	3.17	<0.01
Acetyl-beta-methylcholine	3	3.32	<0.01
PC(14:0/14:0)	1	4.67	<0.01
N1, N1-diethyl-4-[5-(4-nitrophenyl)-1,3-oxazolan-2-yl]aniline	3	4.93	<0.01
(7E)-12-Hydroxy-3-isobutyl-13-methoxy-4,5,8-trimethyl-3,3a,4,6a,9,10,11,12,13,14-decahydro-1H-cycloundeca[d]isoindole-1,15(2H)-dione	3	5.25	<0.01
Theophylline	1	6.69	<0.01
4-amino-1-phenyl-6-(phenylimino)-1,2,5,6-tetrahydro-1,3,5-triazin-2-one	3	7.50	<0.01
Theobromine	1	7.71	<0.01

^*^Log2 Fold change is calculated as BSFL/CON. Values ≥1 indicate higher abundance in the BSFL group; values ≤−1 indicate higher abundance in the control group. ^#^Levels of identification correspond to standard metabolite identification confidence in untargeted LC–MS: Level 1 = confirmed identification with authentic reference standard, Level 2 = putatively annotated compound based on spectral library match, and Level 3 = putatively assigned compound class.

**Figure 2:**
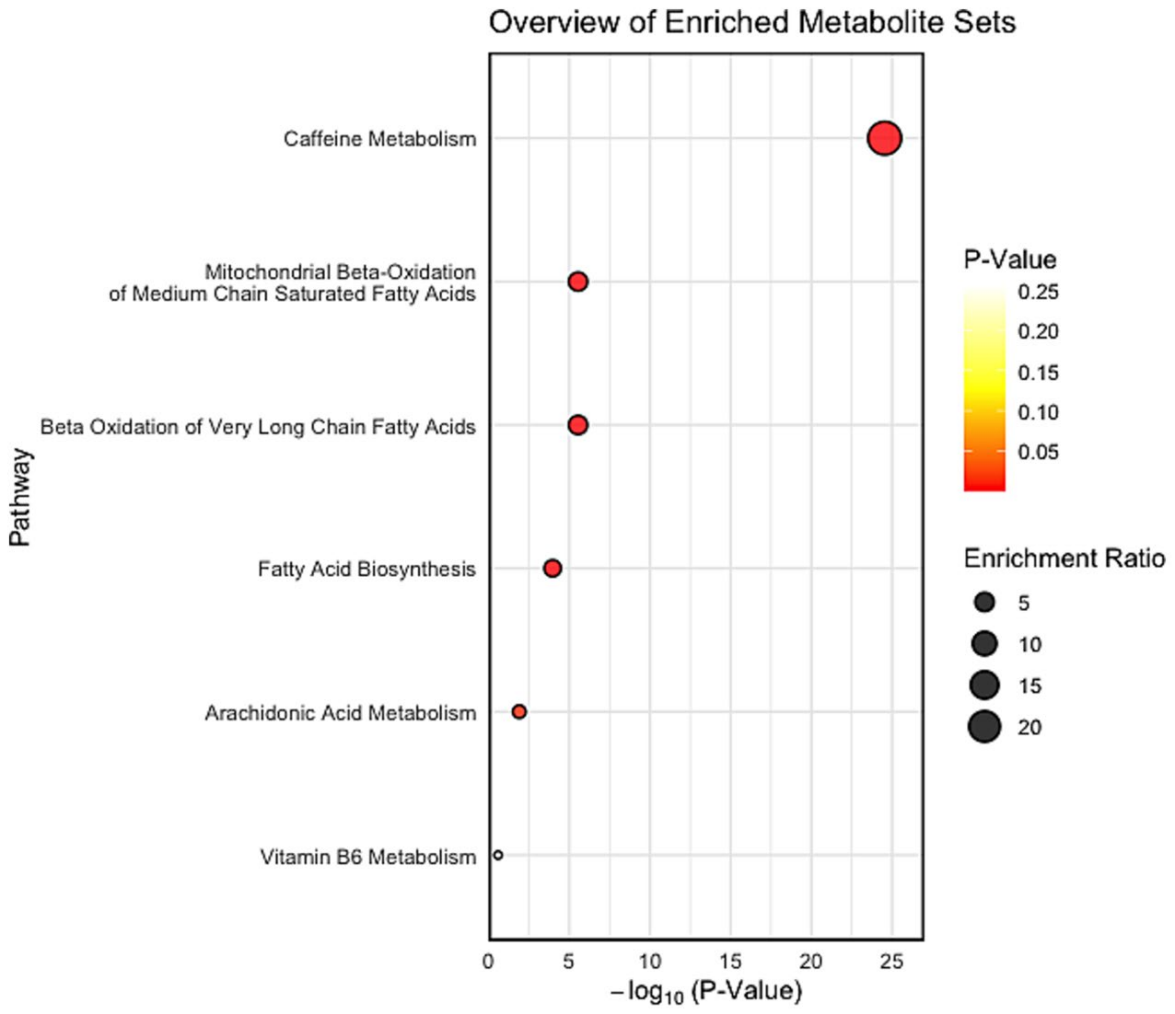
Pathway enrichment analysis highlighting metabolite sets altered in Silkie crossbreed chickens after dietary BSFL inclusion compared with controls.

As shown in Figure 2, pathway enrichment analysis revealed several significantly impacted pathways. Caffeine metabolism showed the strongest enrichment (*p* < 0.001), driven by the detection of theophylline and theobromine. Beta-oxidation of very long–chain fatty acids, mitochondrial beta-oxidation of medium-chain saturated fatty acids and fatty acid biosynthesis were significantly enriched (*p* < 0.001), supported by the presence of lauric acid, myristic acid and related derivatives, including 12-hydroxydodecanoic acid, dodecanedioic acid, and 3-oxodecanoic acid. Arachidonic acid metabolism was enriched at a lower level (*p* < 0.0248), with contributions from PC(34:4), LysoPC(14:0/0:0) and 14-hydroxy-docosahexaenoic acid. In contrast, vitamin B6 metabolism did not reach statistical significance (*p* = 0.258), although pyridoxamine was detected.

### 3.3 Correlations between serum metabolome and growth performance

**Figure 3:**
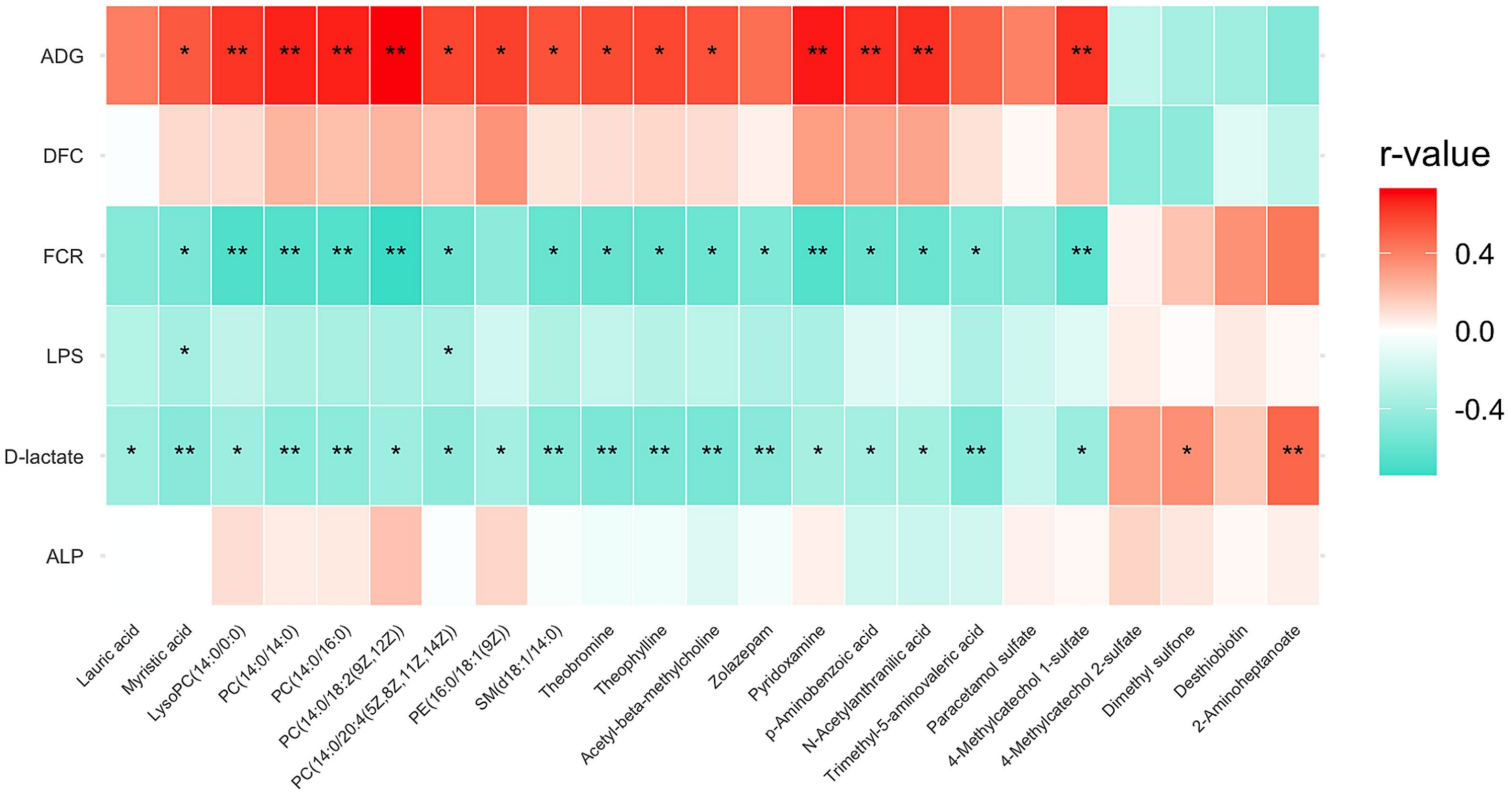
Correlation heatmap of serum metabolites with growth performance and intestinal health metrics. Asterisks indicate statistical significance based on Pearson correlation analysis: *p < 0.05, ** *p* < 0.01. ADG, average daily gain; DFC, daily feed consumption; FCR, feed conversion ratio; LPS, serum lipopolysaccharide; ALP, serum alkaline phosphatase.

To explore the relationships between serum metabolites and physiological traits, a Pearson correlation analysis was performed. As shown in Figure 3, ADG, FCR and D-lactate exhibited the strongest and most frequent correlations with metabolites. Several phosphatidylcholines (PCs), LysoPC(14:0/0:0) and SM(d18:1/14:0) exhibited significant positive correlations with ADG, the main indicator of growth performance (*p* < 0.05), whilest showing significant negative correlations with FCR and D-lactate (*p* < 0.05). Theophylline and theobromine, contributing to caffeine metabolism, showed a positive correlation with ADG and a negative correlation with FCR and D-lactate (*p* < 0.05). In addition, desthiobiotin, 2-aminoheptanoate, 4-methylcatechol 2-sulphate and dimethyl sulphone showed an inverse correlation pattern; however, only 2-aminoheptanoate and dimethyl sulphone were significantly correlated with D-lactate (*p* < 0.01). Notably, only myristic acid and PC(14:0/20:4(5Z,8Z,11Z,14Z)) demonstrated a significant negative correlation with LPS (*p* < 0.05), whilest lauric acid was significantly negatively correlated with D-lactate (*p* < 0.05).

## 4 Discussion

Our previous study demonstrated that dietary BSFL at an inclusion rate of 150 g/kg increased ADG and lowered serum D-lactate, a gut health–associated serum marker (3). To explore the metabolic basis of these effects, the present study investigated how the dietary inclusion of black soldier fly larvae meal influences systemic metabolism in slow-growing Silkie crossbreed chickens.

To explore the metabolic basis of these performance effects, untargeted serum metabolomics was conducted. Clear separation was observed between the BSFL and control birds, supported by 40 significantly altered metabolites. Pathway enrichment indicated shifts in lipid metabolism, fatty acid biosynthesis, β-oxidation, arachidonic acid turnover and xenobiotic metabolism. Although serum profiles represent snapshots of systemic metabolism, these changes suggest potential effects on nutrient absorption, utilisation and metabolic resilience.

BSFL dietary inclusion was associated with elevated serum levels of MCFAs, particularly lauric (C12:0) and myristic (C14:0) acids, which differ metabolically from long-chain fatty acids, such as palmitic acid (22). Lauric acid is preferentially absorbed via the portal vein and rapidly oxidised in the liver, providing a readily available energy source that supports *β*-oxidation and metabolic efficiency (23, 24). These properties offer a plausible metabolic explanation for the improved energy-related performance observed in BSFL-fed birds, including higher ADG and a numerical reduction in FCR. In contrast, longer-chain fatty acids tend to enter the lymphatic pathway and can be incorporated into complex lipids (22). This difference in metabolic routing helps explain why increased dietary MCFA intake was reflected in serum profiles, whereas long-chain fatty acids, such as palmitic acid, did not show measurable changes. Beyond their role in host energy metabolism, MCFAs, such as lauric acid, exert antimicrobial activity in the intestinal lumen, which contributes to modulating gut microbial composition (7, 25). Although gut lumen concentrations were not measured in this study, the observed increase in circulating lauric acid suggests greater dietary availability, which could plausibly translate into higher luminal exposure. Supporting this interpretation, serum lauric acid was negatively correlated with serum D-lactate in BSFL-fed birds, suggesting a potential link between MCFA availability and gut microbial activity. This observation aligns with previous studies reporting that MCFA-rich ingredients support gut microbial balance and gut health (26–29). Whilest direct serum measurements were limited in previous studies, tissue data from broilers supplemented with purified MCFAs or insect oils have shown increased lauric acid incorporation (30–32), supporting the biological plausibility that BSFL meal at practical inclusion levels can enhance lauric acid intake and contribute to both metabolic functional effects and gut health.

BSFL feeding also increased multiple phospholipid and sphingolipid species, including PCs and phosphatidylethanolamines enriched with 14:0 and 16:0 acyl chains and sphingomyelin (SM) d18:1/14:0. The positive associations of these lipids with ADG and the negative associations with FCR and serum D-lactate suggest that lipid remodelling may contribute to more efficient nutrient utilisation and reduced microbial stress. Mechanistically, these lipids are key components of cellular membranes, influencing membrane fluidity, transporter function and lipid raft–mediated signalling (33–35). SM species, in particular, have been linked to epithelial barrier integrity and immune signalling (36). The elevation of these lipids, together with lower D-lactate levels, suggests potential contributions to maintaining epithelial integrity and mucosal stability. Alterations in arachidonic acid–linked PCs and reductions in 14(S)-HDHA may reflect balanced eicosanoid turnover rather than heightened inflammatory activity, consistent with reports that dietary phospholipid remodelling supports nutrient absorption and epithelial function under stress (37–41).

BSFL dietary inclusion also influenced microbial-derived metabolites, including tryptophan-derived indoles, such as indole-3-carboxylic acid, in this study. These microbial indoles are known to act as ligands for nuclear receptors, particularly the aryl hydrocarbon receptor, and have been implicated in maintaining gut barrier integrity, supporting epithelial function and modulating immune homeostasis (42). The elevation of indole-3-carboxylic acid in BSFL-fed birds suggests a potential mechanism by which insect-derived feed may enhance intestinal barrier function and contribute to improved systemic metabolism (43). In addition, several xenobiotic-like compounds, including theobromine, theophylline and paracetamol sulphate, were elevated, likely reflecting the activation of phase I and II detoxification pathways rather than direct exposure, indicating possible associations with enhanced systemic metabolic resilience (44). This finding may explain the negative correlation of theobromine and theophylline with FCR and serum D-lactate. Increased dietary theobromine availability altered intestinal microbiota composition in Lewis rats, reducing potentially pathogenic taxa such as *E. coli* and *Streptococcus* spp., whilest also lowering IgA-coated bacteria. At the same time, theobromine promoted certain Firmicutes and increased butyrate production, pointing to enhanced metabolic resilience and reduced microbial stress (45). Moreover, metagenomic analysis in broilers demonstrated that variation in FCR was associated with differences in gut microbiota composition and functional pathways, although theobromine itself was not identified as a differential metabolites in the study (46). Collectively, these findings suggest that microbial modulation contributes to systemic resilience and feed efficiency. In our study, this was reflected by the negative correlation between elevated serum theobromine and theophylline and lower FCR and D-lactate, linking xenobiotic-like metabolite changes to improved gut health and growth performance in BSFL-fed chickens. Further, BSFL meal contains MCFAs, chitin derivatives, peptides and antioxidant molecules (47–50). These components are plausibly capable of influencing hepatic metabolism, as similar compounds in other animal models have been shown to activate Nrf2-associated antioxidant responses and conjugative enzymes, including glutathione S-transferase and NQO1 (51, 52). Whilest this may help explain the elevated xenobiotic-like metabolites observed in the BSFL-fed birds, direct evidence in chickens is currently lacking.

Some limitations should be considered when interpreting these findings. Untargeted metabolomics offers broad metabolic coverage but is inherently semi-quantitative in the absence of isotope-labelled internal standards (53). Compound annotation relies on public spectral libraries that are not fully optimised for avian species, which may reduce confidence in the identification of some metabolites, particularly those at lower annotation levels (54). Consequently, certain differentially abundant features, including structurally complex or xenobiotic-like compounds, were not explored in detail and may require further chemical validation using authentic standards or targeted approaches. Finally, serum metabolite profiles reflect systemic metabolism and cannot resolve tissue-specific or luminal processes. Future studies integrating targeted metabolomics, tissue-level analyses and microbiota profiling will be necessary to confirm candidate biomarkers and clarify underlying mechanisms.

## 5 Conclusion

Collectively, these findings showed that the dietary inclusion of 150 g/kg BSFL meal induced systemic metabolic changes in slow-growing Silkie crossbreed chickens, particularly affecting lipid and microbial-related metabolites. These changes support energy metabolism via the direct absorption of MCFAs and enhance cellular function and signalling through the remodelling of complex lipids, such as phospholipids and sphingolipids. BSFL supplementation also indirectly influenced metabolism and gut health through microbial-derived metabolites, including indole-3-carboxylic acid, which modulate gut microbial composition and epithelial function. Overall, these metabolic alterations were associated with improved growth performance and reduced serum D-lactate, without evidence of adverse effects, suggesting a beneficial impact of BSFL inclusion at the tested level. Whilest mechanistic conclusions cannot be drawn from metabolomics alone, these results provide a foundation for future studies to validate candidate lipid biomarkers, investigate dose–response effects and assess tissue-level metabolic remodelling in the intestine and liver. The integration of metabolomics with conventional performance and physiological markers offers a comprehensive framework for understanding how insect-derived feed ingredients influence metabolism and gut health in poultry.

## Data Availability

The original contributions presented in the study are included in the article/supplementary material, further inquiries can be directed to the corresponding author.
